# Accurate Microwave Circuit Co-Simulation Method Based on Simplified Equivalent Circuit Modeling

**DOI:** 10.3390/mi14101847

**Published:** 2023-09-27

**Authors:** Sanghyun Kim, Won-Sang Yoon, Jongsik Lim, Sang-Min Han

**Affiliations:** 1 Department of ICT Convergence, Soonchunhyang University, Asan 31538, Chungnam, Republic of Koreajslim@sch.ac.kr (J.L.); 2 Department of Electronic Engineering, Hoseo University, Asan 31499, Chungnam, Republic of Korea

**Keywords:** Computer-Aided Design (CAD), co-simulation, de-embedding, diode models, equivalent circuit models, passive resonant devices, reconfigurable circuits

## Abstract

A new co-simulation method is proposed for active devices and electromagnetic resonant circuits at microwave frequency range. For the measured and extracted device parameters, three steps of equivalent circuit models are processed of the general, simplified, and EM RLC models. To overcome the limited lumped element simulation in an electromagnetic simulator, the simplified equivalent circuit model is established by mathematical computation. The co-simulation procedures are described and experimentally verified for commercial diodes. The application circuit is designed and implemented using the proposed co-simulation method. The experimental results verify that design using the proposed co-simulated method presented excellent agreement for a wideband frequency range of 0–4 GHz, compared with that using a conventional design method. The proposed co-simulation method can be applied to any commercial EM simulation tools without active model error.

## 1. Introduction

Recently, as various wireless and connectivity services have required a compact and multi-functional mobile terminal, composite and complex system design technologies have been rapidly developed, such as active integrated antennas [1], tunable filters [2,3], and reconfigurable systems [4]. These RF front-end circuits and sub-systems are in general designed using RF Computer-Aided Design (CAD) software, which supports accurate, rapid, and low-cost design procedures. Because the CAD tools, a so-called simulator, can provide accurate non-linear property and correct unexpected side effects such as electromagnetic couplings and resonances, it is useful to overcome the limitation of conventional design methods.

Recent RF/microwave simulators are categorized into the electromagnetic (EM) simulator, and the RF circuit simulator. These two kinds of simulations have fundamentally different approaches to solving circuit problems. In general, the EM simulator solves and calculates electromagnetic field, currents, and voltages from the geometric structures of circuit and material properties, which can provide an electromagnetic resonance and field distribution for passive microwave circuits. However, because the EM simulator does not support active device models or any DC bias conditions, it cannot be applied for active circuit design. Meanwhile, the RF circuit simulator supports theoretical equation solvers for specified active and passive device models. Because it works only for previously modeled components, customized designs cannot be utilized, and inter-structure coupling effects are ignored. Therefore, because the two types of simulators have been utilized as alternative tools to each other, and are good for individual device designs, they are not suitable for recent composite integrated devices and sub-systems. Therefore, co-simulation is required for integrated design with both resonant elements and active devices. Conventional co-simulation methods embed a component model in one simulator into the other simulators. While the EM simulated result of a passive device is embedded into a circuit simulator, each result is solved individually, without considering the effect on the EM performance by active components such as resonant frequency variation. An active device is just ideally modeled for EM simulations, for example, a fixed ideal capacitor for a varactor diode, and a short or open circuit for a switch diode, which cannot provide accurate simulation design [5,6].

Therefore, from the requirements of EM and circuit co-simulations, various approaches have been introduced excluding the measurement data importing method provided by commercial software. Most co-simulation approaches utilize importing methods provided by commercial simulation software [7,8,9]. In general, the studies introduce and focus on optimum co-simulation techniques for specific circuit environments such as MMICs [7], PCB loss [8], and a bias circuit [9]. Customized codes are co-simulated with commercial simulation tools in [10,11,12]. Customized coding such as an FDTD and Python can provide precise results compared to the general method provided by commercial tools. However, the device modeling is limited in the specific structure and the co-simulation process requires additional work, even though this method can allow finer precision. Moreover, a manual combination of two simulated results from commercial software was introduced in [13], and an accuracy improvement method was proposed in [14].

The various recent studies have presented excellent improvements and more accurate results. However, from a microwave circuit co-simulation point of view, a few figures of merit should be considered for simulation availability such as active and passive device co-simulation, the resonant effect simulation by active device models, and circuit model filing for design libraries. In addition, the co-simulation method should be applied to every circuit environment for any commercial simulation tools.

In this paper, a new co-simulation method and procedures of EM resonant circuits and active devices are proposed for RF integrated or reconfigurable resonant circuit design applications. An accurate equivalent modeling process is presented for an active component. The extracting and modeling procedures are introduced for active devices in EM simulations. Since active devices at microwave ranges operate very sensitively to operating conditions, such as input RF power, the supplied DC bias, sub-circuits, and substrates, they are not well matched to equivalent circuit models from vendor specifications. In addition, it is difficult to directly apply the complicated circuit model for an EM simulator. The proposed method characterizes the Device Under Test (DUT) from the fundamental circuit models, including parasitic elements, and then simplifies the model from mathematical computations. The simplified modeling approach can provide multiple lumped element simulations in EM simulators over microwave bands. Therefore, from extracting component parameters to EM circuit modeling, active component EM RLC models can be filed in a design library at each bias condition. For complex equivalent circuits with series–parallel composite connections, unexpected bust errata in EM simulations can be eliminated. From the simplified equivalent circuit model, the EM simulation embedding active device models can be performed without EM RLC model error. In addition, the proposed co-simulation method can simulate the passive resonant effect by active device variations using filed device libraries. Furthermore, the proposed method can be a general solution for precise co-simulation of active and passive devices, although a few commercial softwares provide co-simulation options, because it is applicable to any commercial EM simulation tools.

This paper is organized as follows. Section 2 introduces the proposed co-simulation procedure. Section 3 describes a de-embedding calibration and an equivalent circuit modeling of varactor and Schottky diodes to verify the proposed process. Section 4 presents the co-simulated and experimental results of the varactor diode application circuit for various bias conditions. Finally, Section 5 concludes the paper with mention of the applications of the new proposed co-simulation method.

## 2. Co-Simulation Method with Simplified Equivalent Circuit Models

A co-simulation method and design procedure for active devices and passive resonant circuits are introduced. Because a circuit simulator does not support resonant circuits, the active component is modeled and embedded into a commercial EM simulator to co-simulate with passive resonant circuits. An active component is modeled, including parasitic parameters for each bias status, which files the models in an active component library in an EM simulator.

The circuit operating characteristics are measured and extracted from active components [15,16,17]. Based on a general circuit model with parasitic elements, the equivalent circuit composed of R_s_, L_s_, and C_s_ is modeled. Since the equivalent circuit model is established from specifications provided by vendors, specific parameters are required to be replaced or changed to meet specific frequency ranges and bias conditions. Each equivalent circuit is designed for operating conditions such as bias and control voltages. As a varactor diode has variable capacitances according to control voltages, the impedance of the varactor diode should be measured at each control voltage.

The lumped elements of the general equivalent circuit model are mapped to ideal RLCs in a commercial EM simulator. However, since a commercial EM simulator provides limited lumped element simulation, the complicated connection of multiple lumped elements does not match the extracted parameters, even though the EM simulator works. Furthermore, because the ideal RLC units are implemented with rectangular surfaces or cubic structures, and the EM simulator may try to solve through every current flow, the complex series-parallel composite connected configuration of the equivalent circuit makes unexpected errata at high-frequency ranges. Therefore, an additional step is required to make a simplified equivalent circuit model, which from mathematical calculations re-arranges the model to a series-connected single RLC configuration. The simplified equivalent circuit has a series-connected single resistance, inductance, and capacitor.

**Figure 1:**
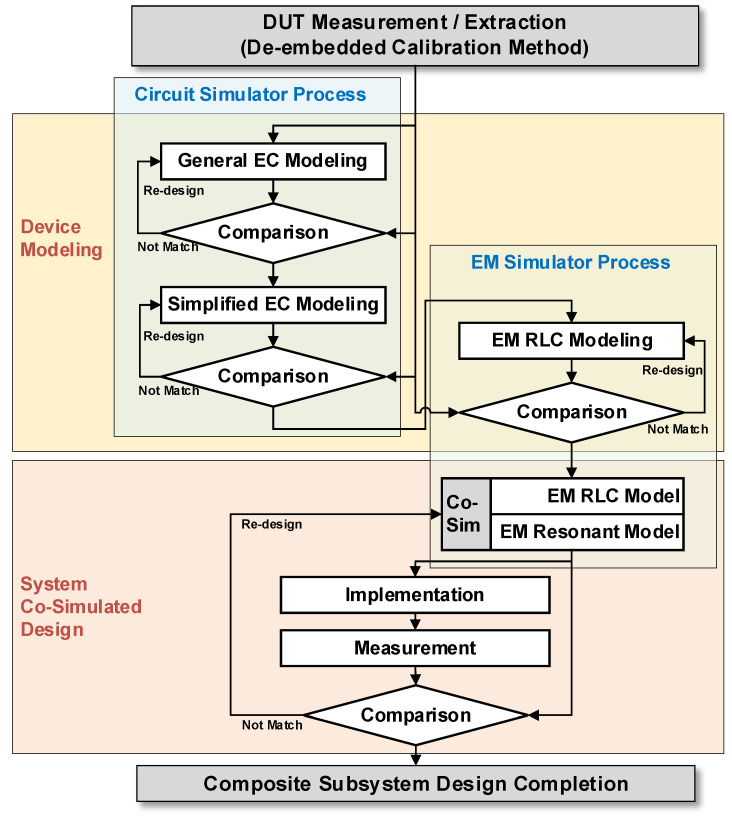
Co-simulation procedures using the simplified equivalent circuit (EC) model.

Then, the simplified equivalent circuit model is mapped to the EM RLC model. From the measurement and extraction step to the final EM RLC modeling step, each parameter should be matched for frequency ranges, and the co-simulated results using the EM RLC models should be matched to implemented design results. The modeling process for the proposed co-simulation method is described in Figure 1.

### 2.1. Parameter Extraction Using De-Embedded Calibration

For the proposed co-simulation method, active component parameters are measured or extracted by a de-embedding calibration method. Because the vendor specification provides ideal and limited operating conditions, specific parameters should be extracted to fit the device and subsystem design conditions.

**Figure 2:**
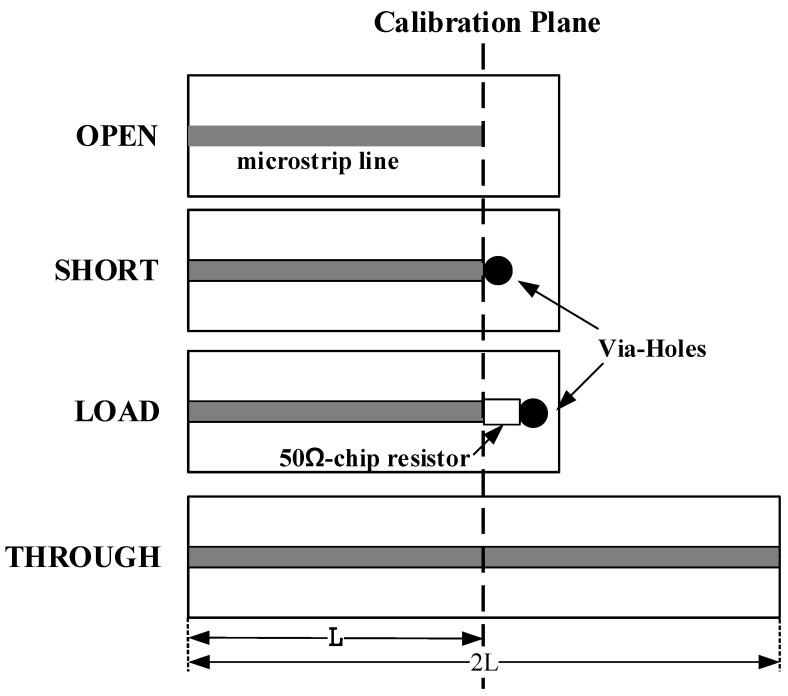
Customized calibration kits of an open, a short, a load, and through circuits for de-embedded calibration.

The de-embedded calibration method is utilized to measure the parameters, including the substrate environment. The customized calibration kit can de-embed feedline and connector effects, while the extracted model contains the electromagnetic effect of the substrate mounting the DUT. The customized calibration kits are presented in Figure 2. Each calibration kit has the same feedline length. The customized calibration kits are utilized for commercial network analyzer calibration. The extracted S-parameters become references compared to the results of equivalent circuit models for each step.

### 2.2. General Equivalent Circuit Modeling

**Figure 3:**
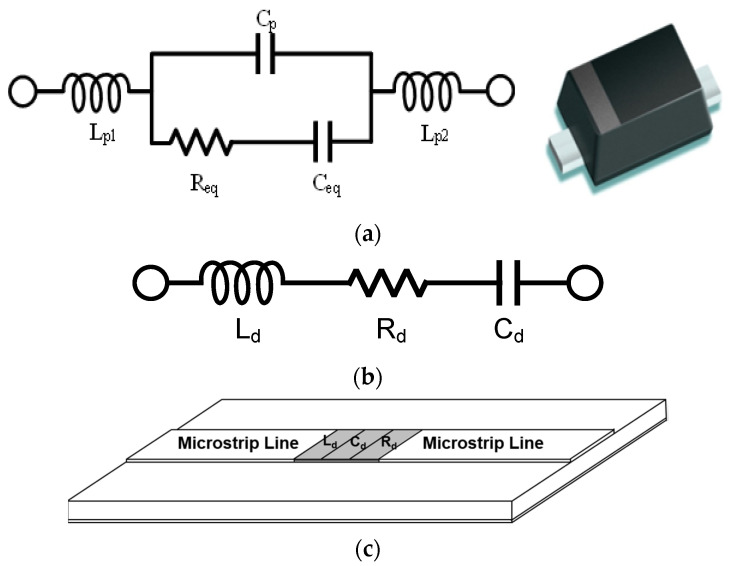
Varactor diode equivalent circuit models. (**a**) General equivalent circuit model and chip photograph. (**b**) Simplified equivalent circuit model. (**c**) EM RLC model with transmission lines in EM simulators.

A general equivalent circuit is implemented from the extracted parameters, as shown in Figure 3a. The circuit model can be achieved by modifying the vendor specifications. Parasitic elements and configuration are provided by the vendor. However, each element value can be determined by comparing the extracted parameters, and the configuration can be re-organized. For the general equivalent circuit model, each element value can be found for each bias condition.

### 2.3. Simplified Equivalent Circuit Modeling

Because the general equivalent circuit model has a complex series-parallel composite connection of lumped elements, it cannot be applied for EM simulation. Recent EM simulators provide ideal simulation options for lumped elements with simple connections [18,19]. The general equivalent circuit can be simplified by mathematical computation from multiple RLCs to single RLC, as shown in Figure 3b. For example, the impedance parallel connected C_p_ and series R_eq_ and C_eq_ can be calculated by the left side of Equation (1). The total impedance can be simplified with the right side of Equation (1) with single R_d_ and C_d_:

(1) $$\left(R_{eq}+\frac{1}{j\omega C_{eq}}\right)∥\frac{1}{j\omega C_{p}}=R_{d}+\frac{1}{j\omega C_{d}}$$

From the formula computation, the series-connected single R, L, and C can be replaced by the general equivalent circuit model. The S-parameter results of the simplified equivalent circuit model need to be matched to the initial extracted parameters and the general equivalent circuit modeled parameters.

### 2.4. EM RLC Modeling

From the simplified equivalent circuit model, the series-connected single R, L, and C is modeled in an EM simulator as presented in Figure 3c. For the EM RLC model, the lumped elements need to connect with transmission lines on both terminals in EM simulators. That is the limitation of EM simulators. Therefore, a minimum length of the extended microstrip line in the EM RLC model should be considered. The EM RLC model parameters should be matched to the extracted, general, and simplified equivalent circuit model parameters. To compare in the same environments, previous models connect microstrip lines on both terminals. When all parameters are matched, the model can be verified for microwave frequency ranges. Figure 3 shows each equivalent circuit models:

## 3. Diode Modeling Using the Proposed Method

### 3.1. Varactor Diode Modeling

The proposed co-simulation method is applied to a varactor diode modeling. The varactor diode of BBY62-02V, the Infineon Technologies with low parasitic elements is utilized for a commercial EM simulator of the AnSys HFSS. The varactor vendor provides a general equivalent circuit model for various packaging types. The vendor provides the series elements of R_s_ = 0.6 Ω, L_s_ = 0.6 nH, and variable capacitance of C_var_ = (2.7–36) pF. From the measured/extracted data of the varactor diode, a general equivalent circuit model is established for each reverse bias of 0, −1, −2, −3, and −4 V, respectively, as shown in Figure 3a. The modeled parameters are fitted to the extracted parameters obtained by de-embedded calibration measurements. The parasitic elements are L_p1_ = L_p2_ = 0.16 nH and C_p_ = 90 fF, while the variable elements for the control voltages of R_eq_ and C_eq_ are summarized in Table 1.

The general equivalent circuit model is converted to the simplified equivalent circuit model with single R, L, and C. To reduce composite connections, the general equivalent circuit elements of C_p_, R_eq_, and C_eq_ in Figure 3a are computed to single R_d_ and C_d_ in Figure 3b, as presented in Equation (1). Two lead inductances of L_p1_ and L_p2_ are merged to L_d_. The simplified element values have L_d_ = 0.32 nH, while the variable elements of C_d_ and R_d_ for each bias condition as presented in Table 2.

**Figure 4:**
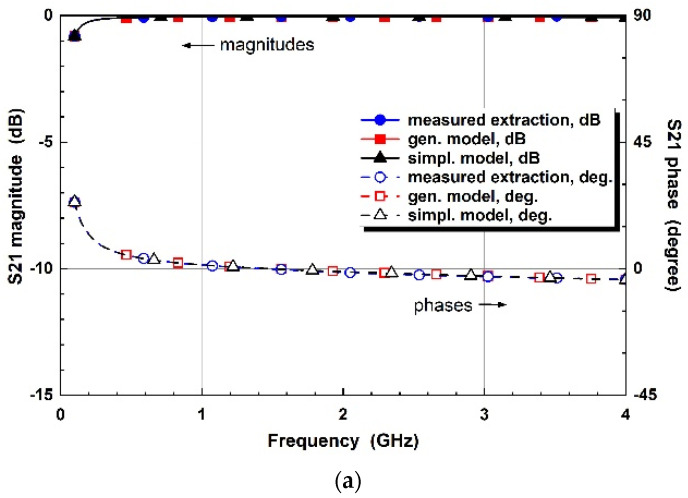

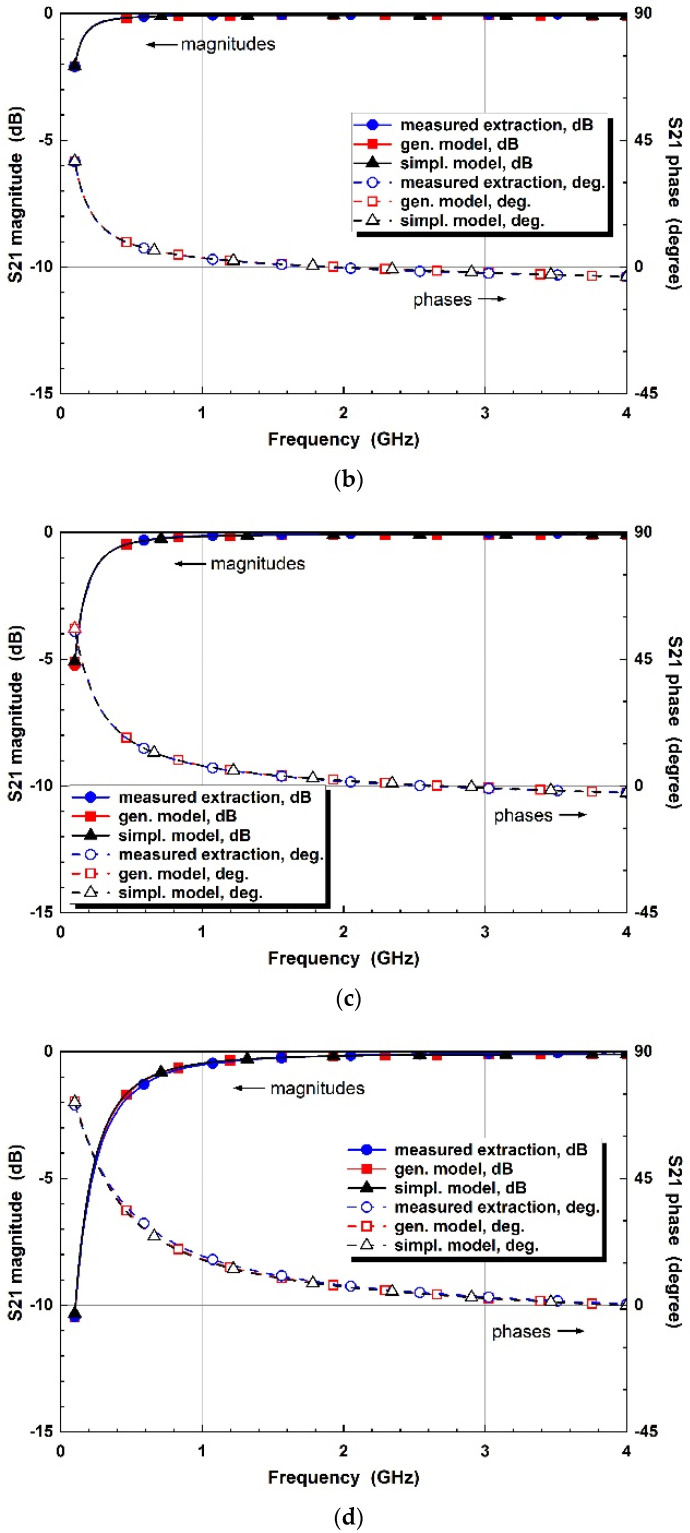

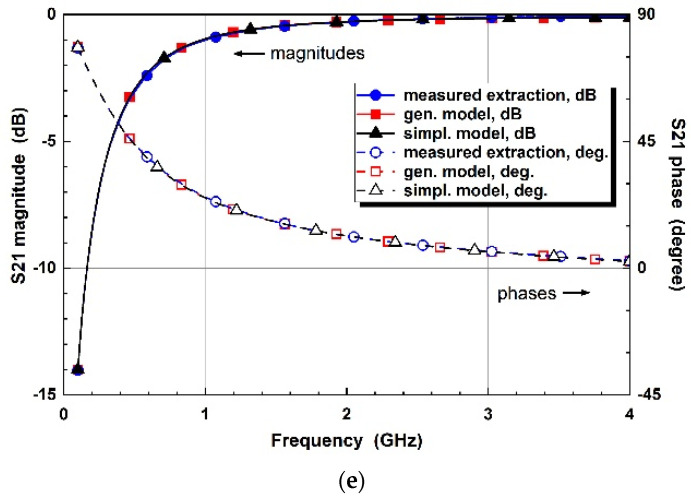
Comparison of varactor diode parameters of the measured extraction, general equivalent circuit models, and simplified equivalent circuit models for control voltages of (**a**) V_R_ = 0 V, (**b**) V_R_ = −1 V, (**c**) V_R_ = −2 V (**d**) V_R_ = −3 V, and (**e**) V_R_ = −4 V.

Two-step modeling in the proposed co-simulation method is evaluated by matching microwave parameters. The cathode of the varactor diode is set to port 1, while the anode is set to port 2 for S-parameter measurements by a vector network analyzer. The verification is performed by comparing the extracted parameters to the simulated models. Figure 4 compares the diode parameters of the measured extraction, general, and simplified equivalent models for each reverse bias. The phase parameters, as well as magnitudes, are compared to verify the equivalence. From the comparison, each parameter is well matched for the 0 to 4 GHz frequency ranges and 0 to −4 V bias conditions.

**Figure 5:**
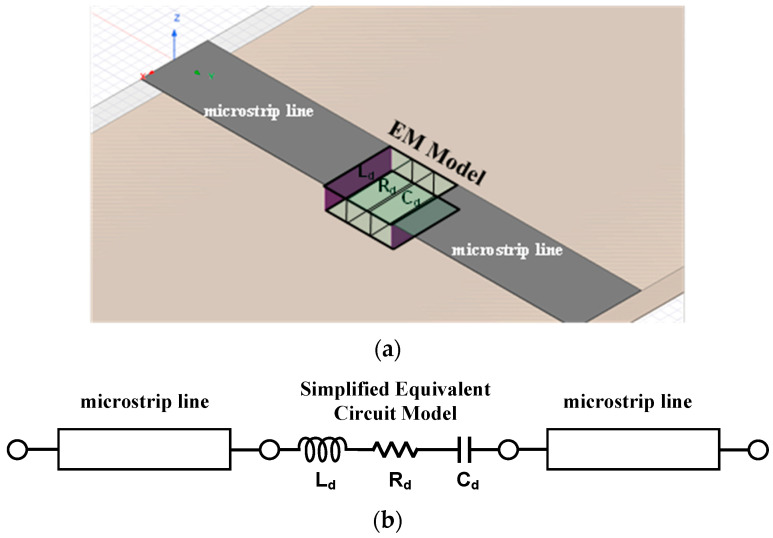
EM RLC modeling. (**a**) Configuration of EM RLC model. (**b**) Reference simulation setup for the extracted parameter modification.

**Figure 6:**
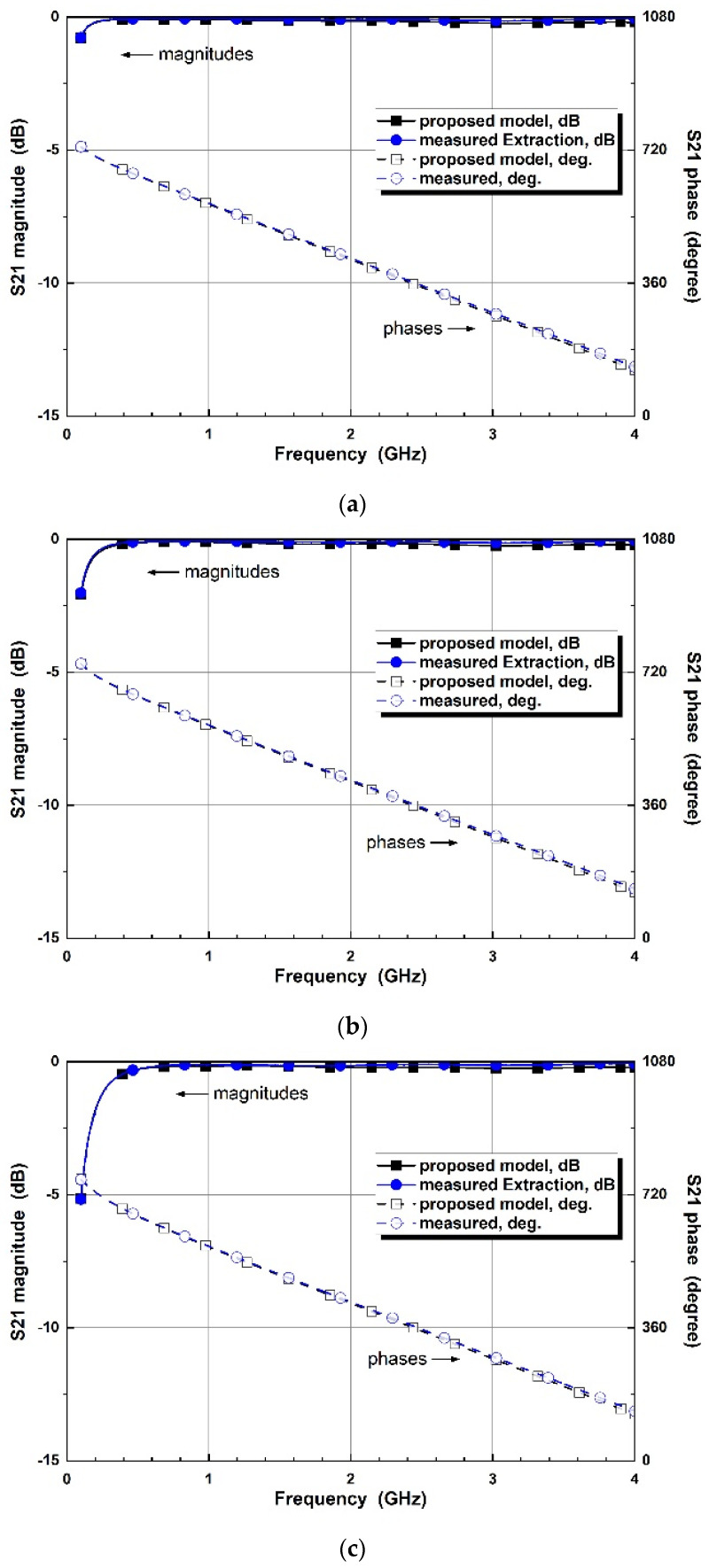

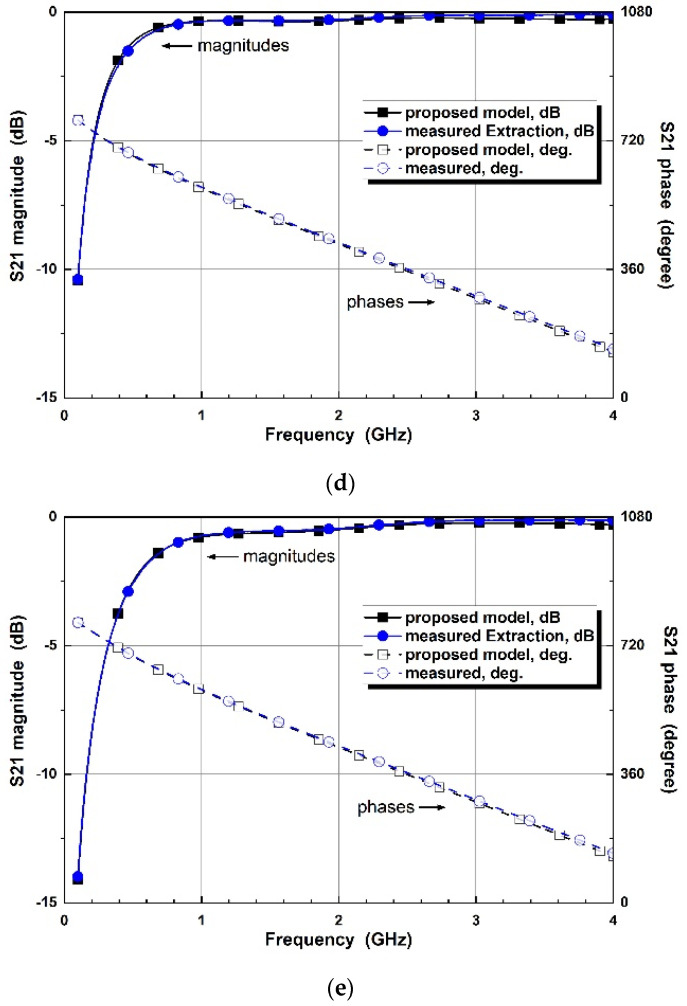
Comparison of varactor diode parameters of the measured extraction and the EM RLC model with transmission lines for control voltages of (**a**) V_R_ = 0 V, (**b**) V_R_ = −1 V, (**c**) V_R_ = −2 V, (**d**) V_R_ = −3 V, and (**e**) V_R_ = −4 V.

The simplified series-connected signal RLC models are mapped for an EM simulator. Because a commercial EM simulator supports the ideal R, L, and C model with transmissions to make simulation ports, the simplified model is implemented with microstrip lines on both terminals of the RLC model. Each microstrip line has a length of 44.4 mm and a width of 2.3 mm on a substrate of RF/Duroid 5880 with a dielectric constant of 2.2 and a thickness of 31 Mils, as shown in Figure 5a; in addition, the extracted parameters are modified with the same microstrip lines for circuit simulations shown in Figure 5b:

Figure 6 compares the extracted and EM co-simulated results for a frequency range of 1–4 GHz. The experimental results of the magnitudes and phases show that the proposed EM RLC model is well matched and verify that the proposed EM RLC models are well established and matched for co-simulation.

### 3.2. Schottky Diode Modeling

An alternative diode model is applied for the proposed modeling and co-simulation method. A Schottky diode is one of the most popular diodes for demodulation, rectification, and frequency mixing applications. However, because it has variable impedances for input RF and microwave power, it is difficult to design impedance matching circuits. In this section, a Schottky diode of HSMS-2850 in Broadcom (Former Avago Technologies, San Jose, CA, USA) is modeled based on the same extraction and modeling method as the varactor diode modeling shown above.

**Figure 7:**
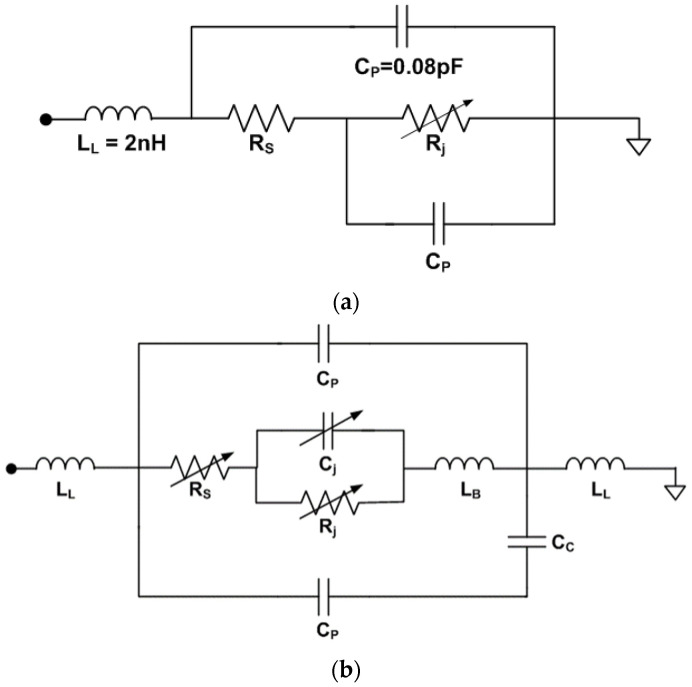
Schottky diode equivalent circuit model. (**a**) Vendor equivalent circuit model. (**b**) Proposed equivalent circuit model.

Figure 7a shows the general equivalent circuit model provided by the vendor specification. From the proposed extraction and measurement method at 2.4 GHz, the model is modified as Figure 7b. The parasitic elements show almost similar patterns, whereas the variable element value of R_s_ and C_j_ can be modified for precise modeling.

The element values are searched for the proposed equivalent model. The fixed parasitic elements have L_L_ = 0.5 nH, L_B_ = 1.0 nH, C_P_ = 0.08 pF, C_C_ = 0.06 pF, while the variable element values for input RF power are summarized in Table 3.

**Figure 8:**
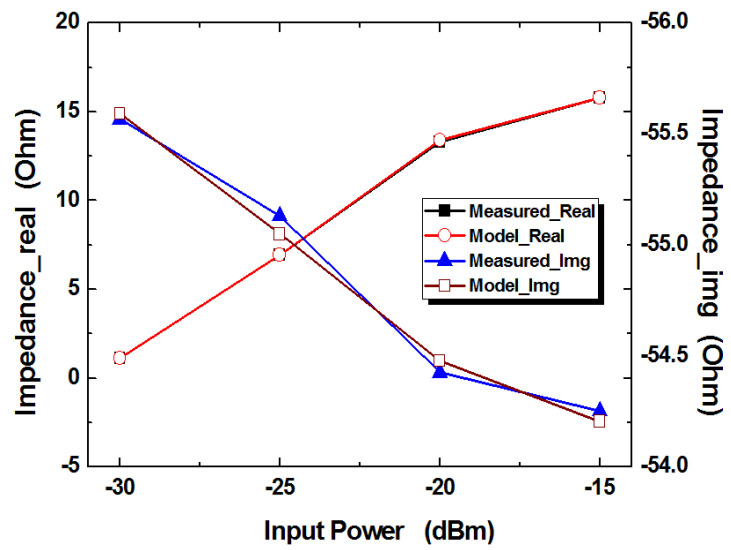
Comparison of measured and modeled impedances for variable RF input power.

For each equivalent circuit model for RF input power, the proposed simplified circuit models are established using the same procedure as Section 3.1. The measured real and imaginary impedances are compared to the proposed models in Figure 8. The experimental verification shows that the proposed modeling method is well matched for measurements.

## 4. Application design and Experiments

**Figure 9:**
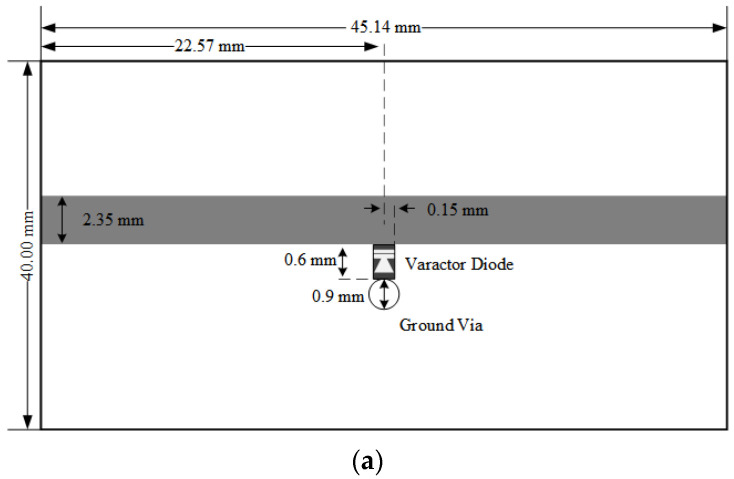

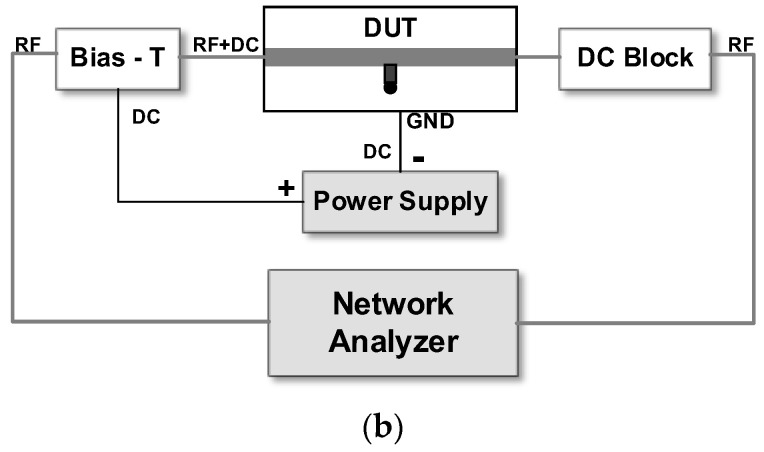
Lowpass filter design using the proposed co-simulation method. (**a**) Design layout. (**b**) Measurement setup.

From the established varactor diode EM RLC models, a variable one-stage lowpass filter was designed to verify whether the proposed method was valid or not. The varactor diode is used as an element connected in parallel. To avoid the effect of other component variations like L’s and C’s parasitic elements, only one element of the diode was used on the transmission lines. Figure 9a shows the design layout with the varactor diode model. Whereas a conventional design uses a single capacitor for variable capacitances, the proposed co-simulation mounts the EM RLC model from the simplified equivalent circuit model. A 50 Ω microstrip line is implemented on a substrate with a dielectric constant of 2.2 and a thickness of 31 Mils, and the varactor diode model is mounted between the microstrip line and a ground via pad. To compare conventional designs, the varactor model is replaced with a single capacitor provided by a vendor specification. For the conventional simulation, the same design setup is applied without replacement of the proposed diode model with an ideal capacitor model. The measurement setup is presented in Figure 9b. Reverse control voltages are supplied to the DUT using a bias-T and a DC block, whereas during calibration process, the bias0T and a DC block are de-embedded.

**Figure 10:**
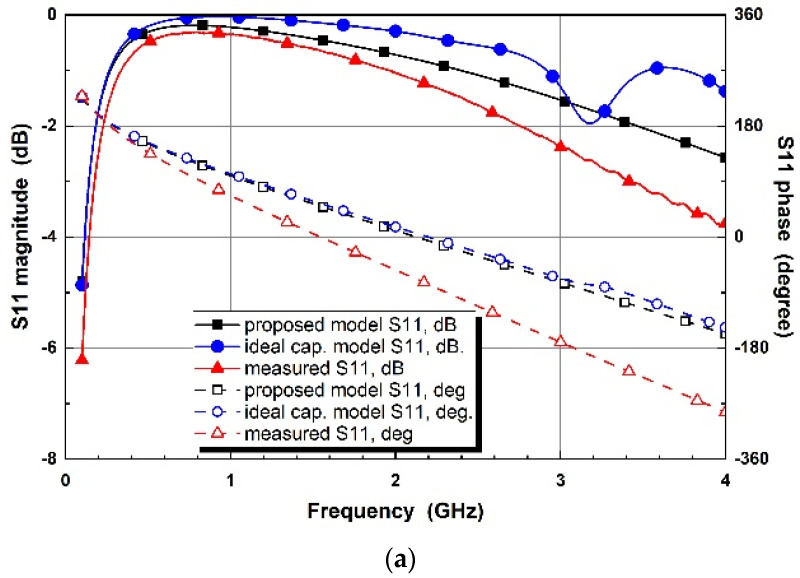

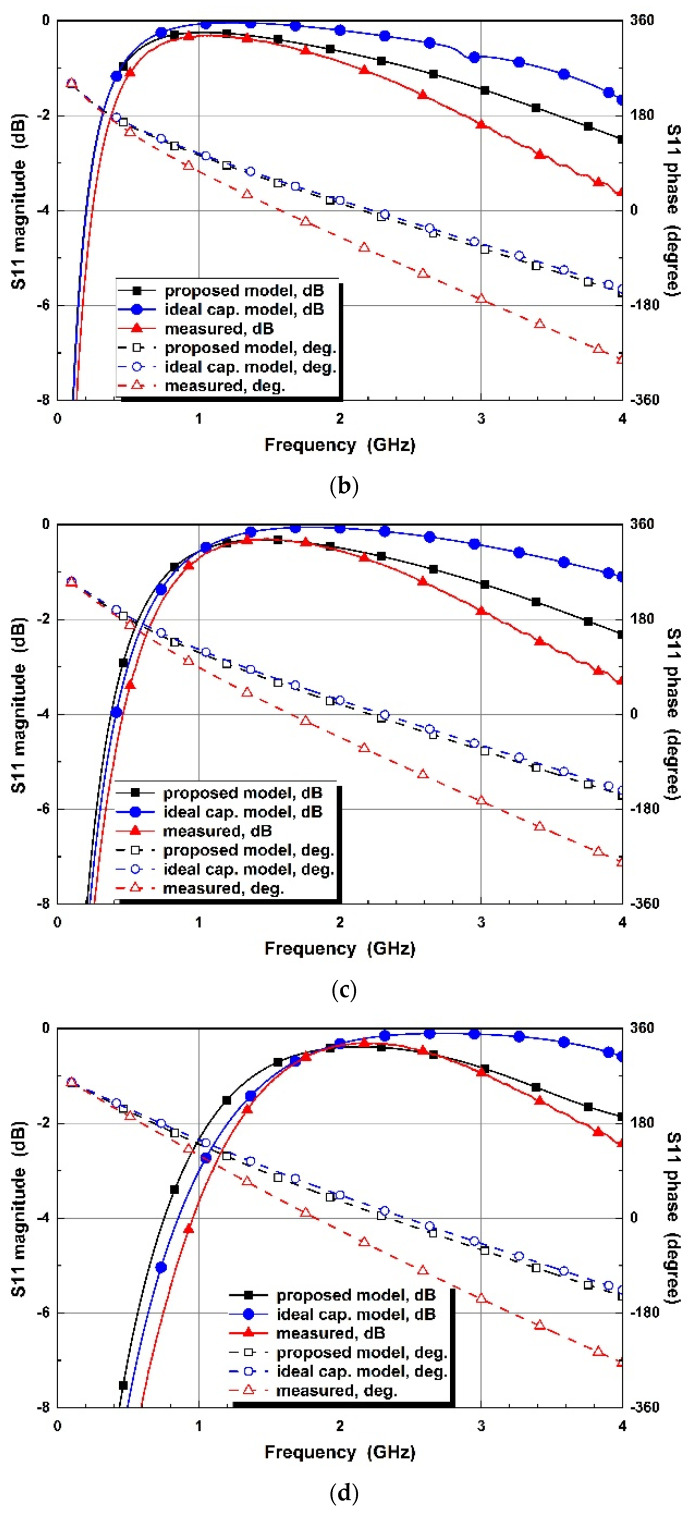

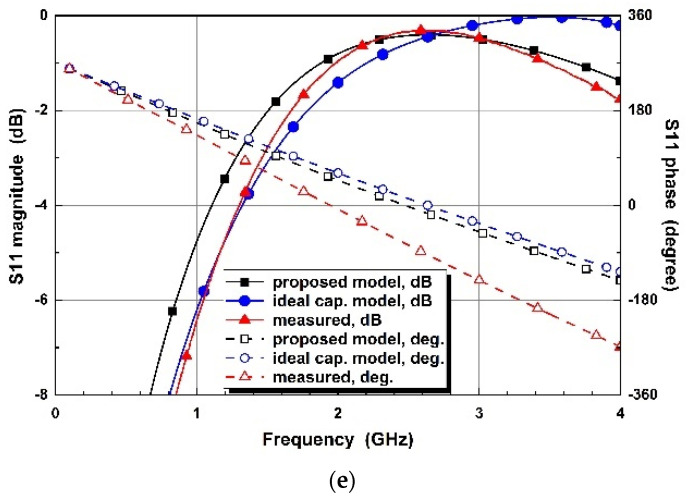
S11 comparison of simulated designs with the ideal capacitors and proposed varactor diode models, and measured results for the control voltages of (**a**) V_R_ = 0 V, (**b**) V_R_ = −1 V, (**c**) V_R_ = −2 V, (**d**) V_R_ = −3 V, and (**e**) V_R_ = −4 V.

Figure 10 presents the simulated and experimental results of S11 and compares two design processes of a conventional single capacitor and a proposed EM RLC model with simplified equivalent circuit with the experimental results. The ideal capacitor model in blue lines shows significant mismatches, whereas the proposed models present excellent matches. The experimental results show that the proposed method achieves more precise active device modeling for EM simulators, compared to conventional ideal capacitance modeling. For each bias status, the design results by the proposed models match the experimental results very well.

**Figure 11:**
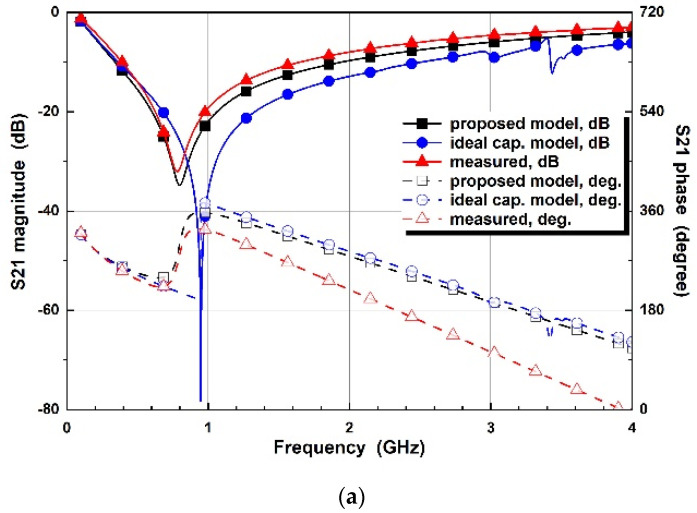

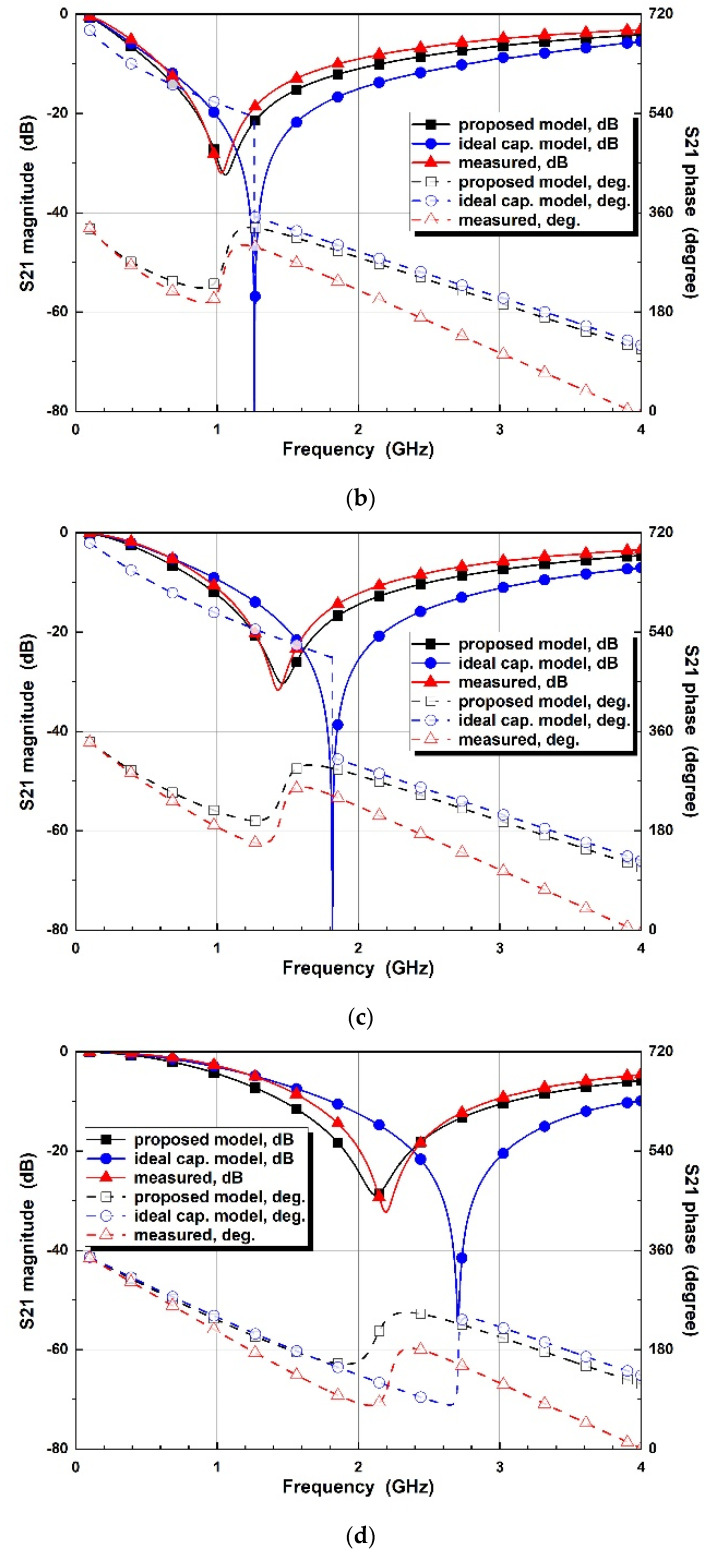

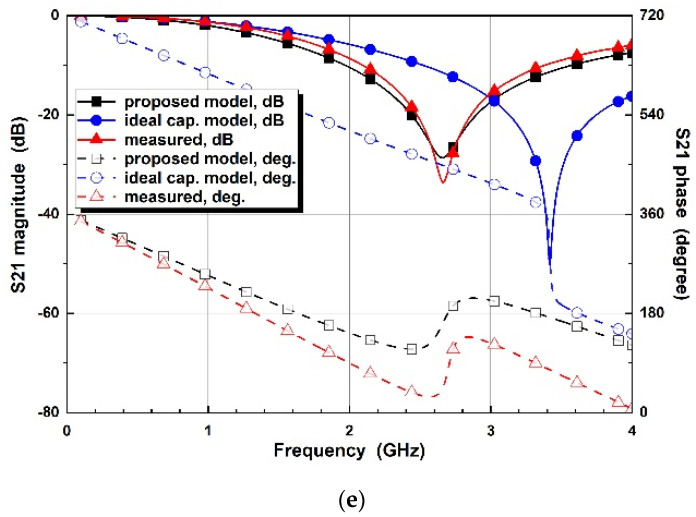
S21 comparison of simulated designs with the ideal capacitors and proposed varactor diode models, and measured results for the control voltages of (**a**) V_R_ = 0 V, (**b**) V_R_ = −1 V, (**c**) V_R_ = −2 V, (**d**) V_R_ = −3 V, and (**e**) V_R_ = −4 V.

Figure 11 presents the S21 comparison of the conventional, proposed designs and the measured results. The pole frequencies of the proposed method are 0.80, 1.06, 1.46, 2.12, and 2.66 GHz for a control voltage of 0, −1, −2, −3, and −4 V, whereas the ideal capacitor model design has pole frequencies of 0.95, 1.27, 1.82, 2.71, and 3.42 GHz, respectively. The measured results for a control voltage of 0, −1, −2, −3, and −4 V show pole frequencies of 0.78, 1.03, 1.43, 2.19, and 2.66 GHz, respectively. The experimental results match the proposed design results better, compared to the conventional design for a wideband frequency range of 0–4 GHz. For the comparisons to the conventional methods of ideal capacitor models, the blue lines show significant mismatches with the measured results, while the proposed model is more closely matched to the measured results.

The proposed work is compared with recent co-simulation studies in Table 4. Each referenced study has advantages for specific design and co-simulation. This proposed work presents a resonant effect and library filing availability with active and passive device co-simulation environment using any commercial EM simulation tools.

## 5. Conclusions

A new co-simulation method is proposed with a simplified equivalent circuit model for active devices and passive resonant circuits. From the simplified modeling technique, the limitations of RLC models in commercial EM simulators could be overcome. The active device modeling procedure is introduced for the general and simplified equivalent circuit models to EM RLC models. By eliminating complex series and parallel configuration, the ideal lumped element simulation with a single series RLC model can be achieved in EM simulations. From the experimental results, the proposed models show results that match the extracted reference parameters well for a wideband frequency range of 0–4 GHz. The verified EM RLC model can be filed in a design library for various applications. The proposed co-simulation method can be applied as a general co-simulation model using any commercial EM software with active and passive resonant circuit models. In addition, the established active device circuit model can be filed as a design library. Because the proposed model is established from the de-embedding measured extraction, it can be applied to a very high frequency region. However, the measured error and the commercial simulator performance for the EM model should be considered. From the proposed co-simulation method, integrated devices and subsystems can be efficiently designed for versatile microwave applications.

## Figures and Tables

**Table 1:** Variable element values of the general equivalent circuit model.

Ctrl Voltage (V)	Variable Resistance, R_eq_ (Ω)	Variable Capacitance, C_eq_ (pF)
0	0.58	35.9
−1	0.78	20.4
−2	1.02	10.6
−3	1.24	5.0
−4	1.25	3.15

**Table 2:** Variable element values of the general equivalent circuit model.

Ctrl Voltage (V)	Variable Resistance, R_d_ (Ω)	Variable Capacitance, C_d_ (pF)
0	0.58	36.0
−1	0.78	20.5
−2	1.02	10.7
−3	1.24	5.10
−4	1.25	3.24

**Table 3:** Variable element values of the Schottky diode model for RF input power.

RF Power (dBm)	R_s_ (Ω)	R_j_ (Ω)	C_j_ (pF)
−30	0.65	11.30	0.694
−25	8.10	10.50	0.700
−20	16.40	9.80	0.708
−15	19.40	9.00	0.712

**Table 4:** Comparison of the recent co-simulation approaches.

	Co-sim. Method	Final sim. Type ^1^	res. cct Co-sim. ^2^	Library avail ^3^	Limit/Demerit
[7]	commercial SW application tool	C	N	N	specific model in MMIC
[8]	commercial SW application tool	E	Y	N	specific circuit model
[9]	commercial SW application tool	C	N	N	introduce co-sim process in ADS
[10]	customized HIE-FDTD calculation	E	Y	N	no active device sim, customized coding required
[11]	ADS-Python co-sim	C	N	Y	extra coding required
[12]	physical model-based circuit sim and FDTD EM sim	customized (FDTD)	Y	Y	extra mathematical eq. and calculation
[13]	TCAD to EM co-sim	E	N	N	combine two independent simulations
[14]	improve accuracy for conventional co-sim method	C	N	N	model and solve for every specific circuit
this work	active device equivalent modeling in an EM simulator	E	Y	Y	modeling steps

^1^ Simulation type to yield final simulated results (C: Circuit simulation, E: EM simulation); ^2^ Co-simulation availability for passive resonance effect by active device variations; ^3^ Circuit model filing availability for a design library.

## Data Availability

Not applicable.

## References

[1] Chang K., York R.A., Hall P.S., Itoh T. (2002). Active integrated antennas. IEEE Trans. Microw. Theory Tech..

[2] Tsai H.-Y., Huang T.-Y., Wu R.-B. (2016). Varactor-tuned compact dual-mode tunable filter with constant passband characteristics. IEEE Trans. Compon. Packag. Manuf. Technol..

[3] Latip M.A.A., Salleh M.K.M., Pasya I. Tuning circuit using varactor diode for tunable bandstop resonator. Proceedings of the 2011 IEEE Symposium on Wireless Technology and Applications (ISWTA).

[4] Lee S.-J., Yoon W.-S., Han S.-M. (2019). Planar beam steerable parasitic array antenna system design based on the Yagi-Uda design method. Int. J. Antennas Propag..

[5] Han S.-M., Lim J., Ahn D., Yoon W.-S. A study of active element characterization by de-embedded calibration measurements. Proceedings of the URSI GASS 2017.

[6] Kim S., Lim J., Han S.-M. Co-simulation method using active device models in microwave simulators. Proceedings of the International Symposium on Antennas and Propagation.

[7] Fu M., Jiang N., Bornemann J., Feng Q. (2023). Accurate simulation of high-gain MMIC amplifiers with microstrip-type transistors. IEEE Trans. Comput.-Aided Des. Integr. Circuits Syst..

[8] Kim K., Hwang H., Nah W. An EM-circuit co-simulation model to predict insertion loss in a busbar-PCB type EMI filter. Proceedings of the IEEE International Joint EMC/SI/PI and EMC Europe Symposium.

[9] Arira M.N., Alam B.R. Co-simulation EM MoM—HB of bias network and the integration of AlGaN/GaN HEMT RF amplifier for S-band operation. Proceedings of the 2022 International Symposium on Electronics and Smart Devices.

[10] Mou C., Chen J., Peng H. (2022). Electromagnetic–thermal co-simulation of planar monopole antenna based on HIE-FDTD method. Electronics.

[11] Yang S., Khusro A., Li W., Vaseem M., Hashmi M., Shamim A. (2022). Optimization of ANN-based models and its EM co-simulation for printed RF devices. Int. J. RF Microw. Comput.-Aided Eng..

[12] Zeng H., Tang Y., Duan X., Chen X. (2019). A Physical model-based FDTD field-circuit co-simulation method for Schottky diode rectifiers. IEEE Access.

[13] Panda S.R., Fregonese S., Deng M., Chakravorty A., Zimmer T. (2020). TCAD and EM co-simulation method to verify SiGe HBT measurements up to 500 GHz. Solid State Electron..

[14] Tian C.-Y., Shi Y., Shum K.M., Chan C.H. (2020). Wave equation-based discontinuous Galerkin time domain method for co-simulation of electromagnetics-circuit systems. IEEE Trans. Antennas Propag..

[15] Ikuta K., Umeda Y., Ishii Y. (1995). Measurement of high–frequency dielectric characteristics in the mm–wave band for dielectric thin films on semiconductor substrates. Jpn. J. Appl. Phys..

[16] Hamdoun A., Roy L., Himdi M., Lafond O. (2015). Characterisation and analytical modeling of GaN HEMT-based on varactor diodes. Electron. Lett..

[17] Han S.-M., Lee S.-J., Kim K., Lim J., Ahn D. (2014). Design considerations for complicated antenna systems on a simple de-embedding method. IEEE Antennas Wirel. Propag. Lett..

[18] AnSys Ltd. (2023). AnSys High Frequency Simulation Software Manual. http://snsys.com.

[19] Keysight Technologies (2023). Pathwave Advanced Design System Manual. http://keysight.com.

